# Identification of traits underpinning good breadmaking performance of wheat grown with reduced nitrogen fertilisation

**DOI:** 10.1002/jsfa.12848

**Published:** 2023-07-26

**Authors:** Peter R. Shewry, Abigail J. Wood, Kirsty L. Hassall, Till K. Pellny, Andrew Riche, Abrar Hussain, Zhiqiang Shi, Ellen F. Mosleth, Mark Charlton, Mervin Poole, Stuart Jones, Keith Newton, Simon Penson, Gary Tucker, Simon Griffiths, Malcolm J. Hawkesford

**Affiliations:** ^1^ Rothamsted Research Hertfordshire UK; ^2^ Department of Biosciences COMSATS University Islamabad Sahiwal Pakistan; ^3^ Nofima AS Ås Norway; ^4^ Allied Technical Centre Berkshire UK; ^5^ Heygates Ltd Bugbrooke UK; ^6^ Warburtons Ltd. Bolton UK; ^7^ Whitworth Bros. Ltd. North Yorkshire UK; ^8^ ADM Milling Limited Corby UK; ^9^ Campden BRI Gloucestershire UK; ^10^ John Innes Centre Norwich UK

**Keywords:** wheat, breadmaking, nitrogen, fertiliser, gluten proteins, grain protein deviation

## Abstract

**Background:**

Nitrogen fertiliser is the major input and cost for wheat production, being required to support the development of the canopy to maximise yield and for the synthesis of the gluten proteins that are necessary for breadmaking. Consequently, current high‐yielding cultivars require the use of nitrogen fertilisation levels above the yield optimum to achieve the grain protein content needed for breadmaking. This study aimed to reduce this requirement by identifying traits that allow the use of lower levels of nitrogen fertiliser to produce wheat for breadmaking.

**Results:**

A range of commercial wheat genotypes (cultivars) were grown in multiple field trials (six sites over 3 years) in the UK with optimal (200 kg Ha^‐1^) and suboptimal (150 kg Ha^‐1^) application of nitrogen. Bulked grain samples from four sites per year were milled and white flours were baked using three types of breadmaking process. This identified five cultivars that consistently exhibited good breadmaking quality when grown with the lower nitrogen application. Chemical and biochemical analyses showed that the five cultivars were characterised by exhibiting grain protein deviation (GPD) and high dough elasticity.

**Conclusions:**

It is possible to develop novel types of wheat that exhibit good breadmaking quality by selecting for GPD and high dough strength. © 2023 The Authors. *Journal of The Science of Food and Agriculture* published by John Wiley & Sons Ltd on behalf of Society of Chemical Industry.

## INTRODUCTION

Nitrogen (N) is the major mineral that determines crop yield, being essential to ‘build’ a canopy and maximise the capture of carbon. However, N is also an important determinant of grain quality, particularly in wheat. This is because it is required for the synthesis of grain proteins, with the gluten proteins forming the major grain protein fraction in wheat. The vast majority of wheat produced globally, including 40% of the wheat produced in the UK, is used for food production, particularly for making bread and other baked products (including cakes and biscuits). Bread wheat is also used to make Asian noodles and as a functional ingredient in many processed foods. Gluten proteins are essential for these uses, providing viscoelastic properties to dough. Consequently, the content and quality of the grain proteins affect the processing quality, with a minimum of 13 g kg^−1^ protein (2.28 g kg^−1^ N) forming the basis of contracts for premium protein levels encouraged to support a range of grists in the UK.

Nitrogen is the largest input in intensive wheat production and the major cost for farmers, with a high environmental footprint due to the energy requirement for fertiliser production. High levels of application may also result in pollution of groundwater as only about 63%–78%, depending on the genotype, of applied N (at 200 kg Ha^‐1^, less at higher N applications) is taken up by the crop^1^ of which about 80%–85% is transported into the grain.^2^ Consequently, unless this efficiency can be improved, the minimum amount of N required to produce 8 t of wheat per hectare (the current UK average yield) at 13 g kg^−1^ protein is about 230 kg N Ha^‐1^. This is above the optimum N application required for yield, which is about 200 kg N Ha^‐1^ in the UK,^3^ and farmers may therefore need to apply up to 50 kg Ha^‐1^ additional N to achieve the required protein content.

It may be possible to reduce this requirement, to a limited extent, by optimising the efficiency of N uptake and utilisation. However, a more viable long‐term solution is to develop new types of wheat and processing systems, which will allow the use of lower N inputs by farmers and lower protein contents for bread making. This will require an increase in the stability and functionality of the gluten proteins, and/or the identification and exploitation of other quality‐related components. This will not only reduce the cost and energy footprint of production but also reduce the energy requirement for dough mixing.

Gluten proteins account for over half of the total grain proteins, with the proportion increasing with greater N application. They are broadly divided into two groups, the monomeric gliadins, which confer viscosity and extensibility to dough, and the polymeric glutenins, which confer elasticity (strength), which is the major requirement for breadmaking. One group of glutenin proteins, the high molecular weight (HMW) subunits, is particularly important, with allelic variation in their composition being related to differences in dough strength. These effects appear to be mediated by direct effects on the size distribution of the glutenin polymers, with ‘good quality’ subunits being associated with increased proportions of large glutenin polymers. We therefore have a good understanding of the molecular basis for the differences in quality associated with allelic variation in the HMW subunits and other gluten proteins.^4^, ^5^

Increasing the grain N results in a higher content of total gluten proteins but there are differential effects on different protein types, with most studies showing increased proportions of monomeric gliadins and decreased proportions of glutenins,^6^, ^7^, ^8^, ^9^, ^10^ leading to increased dough extensibility. However, Pechanek *et al*.^11^ showed that the effect of N on grain protein composition differed between varieties.^11^ Less is known about the effects of nutrition on the glutenin fraction, either on the proportions of the individual subunits or on the size distribution of the glutenin polymers. Thus, both increases^12^ and decreases^11^ in the proportions of HMW subunits have been reported whereas other studies showed differential effects of N on glutenin polymers and processing properties in cultivars with different HMW subunit alleles.^7^, ^13^

The protein content of wheat correlates with functionality within certain limits. Testing for protein content is rapid and cost‐effective, whereas tests for protein functionality are more time consuming. Consequently, protein content has become the major criterion used for trading bread making wheat. However, the functional properties are known to differ between varieties and grain samples and different wheat samples will often be blended to achieve flours with the desired functionality. The emphasis on protein content not only has significant cost implications for growers and processors (as discussed above) but is also limited in value as high protein content does not guarantee the quality of the flour produced from it.

We have therefore compared the breadmaking quality of 40 bread wheat lines grown at high (250 kg N Ha^‐1^) and suboptimal (150 kg N Ha^‐1^) inputs of N fertiliser, determining their breadmaking performance in commercial laboratories and identifying traits related to good performance at lower N input.

## MATERIALS AND METHODS

### Field trials

Field trials with 40 genotypes and cultivars of bread wheat (Supporting Information, Table S1) were carried out at six sites in the UK. These were Rothamsted Research (Harpenden, Hertfordshire, 51° 48′ 19.79″ N, 0° 21′ 11.39″ W) and the experimental farms of Agrii (Throw's Farm, Essex, 52° 10′ 19.2′’ N, 0° 17′ 2.4′’ E; 52° 10′ 55.2′’ N, 0° 17′ 2.4′’ E; and 52° 11′ 13.2′’ N, 0° 15′ 39.6′’ E in 2015–2016, 2016–2017, 2017–2018, respectively) and four UK wheat breeding companies: Limagrain (Woolpit, Suffolk, 52° 13′ 39.18′’ N, 0° 52′ 45.03′’ E), KWS (Thriplow, Hertfordshire, 52° 5′ 49.866′’ N, 0° 6′ 18.7452′’ E), Saaten Union (Newmarket, Suffolk, 52° 9′ 39.6′’ N, 0° 27′ 39.6′’ E) and DSV (Wardington, Oxfordshire, 52° 06′ 39.9″ N 1° 18′ 23.6″ W, 52° 06′ 45.6″ N 1° 17′ 17.3″ W, and 52° 06′ 20.0″ N 1° 20′ 35.5″ W, in 2015–16, 2016–2017 and 2017–2018, respectively). All 40 genotypes were grown twice (2015–2016 and 2016–17) and a subset of 30 genotypes for a third year (2017–2018) (Supporting Information, Table S1). All lines (spring and winter type) were planted in October and each trial comprised plots of 6 × 1.5 m with a seed rate of 250 m^2^. A split‐plot design was used consisting of three replicate blocks with two levels of N fertilisation applied to main plots. These were 150 kg N Ha^‐1^ (N150) and 250 kg N Ha^‐1^ (N250) as ammonium nitrate, generally as split applications of 50:50:50 kg N Ha^‐1^ and 50:150:50 kg N Ha^‐1^ in March, April, and May, with precise timings based on local conditions. All individual subplots also received 40 kg S Ha^‐1^ while other agronomic treatments were standard for the sites. Yields were converted to tonnes/Ha.

### Grain analyses

White flour was produced from 20 kg samples of grain using a Bühler Laboratory Flour Mill MLU 202 at Campden BRI according to a Campden BRI standard procedure. Where replicates were pooled for analysis, equal weights of grain from each replicate were blended thoroughly prior to milling. The Hagberg falling number was determined by the trial providers on the grain harvested from their own sites using their in‐house systems. The N contents of all grain and flour samples were determined by near‐infrared spectroscopy (NIRS) and converted to protein by the factor ×5.7. Grain protein deviation (GPD) was calculated as described by Mosleth *et al*.^13^

Flour water absorption was measured using a Brabender Farinograph and dough extensibility and resistance of the dough using a Brabender Extensograph according to the Cereal and Cereal Applications Testing working group (CCAT) Methods No. 4 and No 3, respectively.

### Breadmaking

Breadmaking was carried out by six milling and baking companies using their standard in‐house test baking procedures with three different breadmaking processes: Chorleywood Bread Process (CBP) (three companies), spiral (two companies), and traditional fermentation (one company). A range of parameters (Supporting Information, Table S2) were measured using in‐house procedures to assign ‘breadmaking quality’ scores to the flours.

### Size‐exclusion high‐performance liquid chromatography

Size‐exclusion high‐performance liquid chromatography (SE‐HPLC) was used to determine the polymer size distribution of proteins extracted from white flour samples. The analysis was performed according to the Profilblé method developed jointly by ARVALIS and l'Institut National de Recherche Agronomique.^15^ Flour (160 mg) was mixed with 20 mL 1% (w/v) SDS in 0.1 mol L^‐1^ phosphate buffer (pH 6.9), sonicated (Misonix Microson XL2000, Qsonica, LLC, Newtown, CT) to solubilize the polymeric gluten proteins, and then centrifuged for 10 min at 4500 x g. An aliquot of the supernatant was sealed in an HPLC vial ready for analysis. Size‐exclusion high‐performance liquid chromatography was conducted using a Jasco (Jasco (UK) Ltd, Great Dunmow, UK) system operating with a TSK gel G 4000SW column (30 cm x 7.5 mm) and a TSK gel SK guard column (7.5 cm x 7.5 mm). The flow rate was 0.7 mL min^‐1^, and detection was performed at 214 nm. Samples from the three biological replicates were pooled prior to analysis. The chromatograms were integrated using a combination of automated algorithms and manual rules developed as part of the Profilblé method. Peak ratios were calculated as reported by Millar.^16^

### Statistical analyses

Yield, grain N, and GPD were analysed with a linear mixed model estimated via restricted maximum likelihood (REML) with random terms given by site/year/block/main plot/plot and fixed terms given by the three‐way term cultivar * N * time. Due to the imbalance in the fixed effects (partial confounding between cultivar and time), terms were sequentially dropped according to the approximate (Kenward–Roger) F‐statistic until all terms remaining in the model were significant at the 5% level.

R/E, which was determined on flour from pooled replicates and sites, was analysed via a linear mixed model with fixed model given by Variety * N and random model given by year and interactions between year and Variety and N.

Analysis of SE‐HPLC variables was carried out using a linear mixed model but because this was restricted to the low N samples only, the fixed model consisted of only genotypic differences and the random model was given by year/variety.

To compare the selected cultivars to the others formally, a structured treatment comparison was included in the linear mixed effects model. Specifically, the term ‘Selection’ compares the average response of the selected cultivars to the average response of the non‐selected cultivars. Non‐selected compares the response between the non‐selected cultivars; selected compares between Crusoe, Gallant, Rumor, Nelson, Genius, and Mv Lucilla. These models were restricted to the low N samples only. Where appropriate these terms were tested for an interaction with time (Yield, GrainN, and GPD). Analysis was carried out in Genstat, 22nd edition.

## RESULTS

### Selection, growth and grain protein content of genotypes

Forty wheat genotypes (Supporting Information, Table S1) were selected to compare their grain composition and processing quality under high (250 kg Ha^‐1^, called N250) and suboptimal (150 kg Ha^‐1^, called N150) N fertilisation. All were winter type unless otherwise stated. They included nine current UK breadmaking cultivars (five classed as group 1 and four as group 2) by UK Flour Millers (UKFM) and two feed cultivars (UKFM group 4), four UK spring cultivars, eight older UK cultivars (including Hereward and Soissons which were favoured in the past by processors), two Hungarian high protein cultivars, five German cultivars (four of which were bred for breadmaking at low protein content), six French cultivars (including two hybrid cultivars), one Danish cultivar and three mutant lines of the UK spring cultivar Paragon (with the Rht2 dwarfing gene, Stay Green, and 1BL/1RS translocation, respectively). All cultivars were grown in replicated randomised trials on six sites for 2 years, with 30 selected cultivars being grown on all sites for a third year.

Detailed analyses were carried out on all 40 cultivars from year 1 but on 30 cultivars and 20 cultivars from years 2 and 3, respectively. The selection of cultivars for growth in year 3 and for detailed analysis in years 2 and 3 was based on their adaptation to UK growth conditions and their processing performance in the previous year or years.

**Figure 1:**
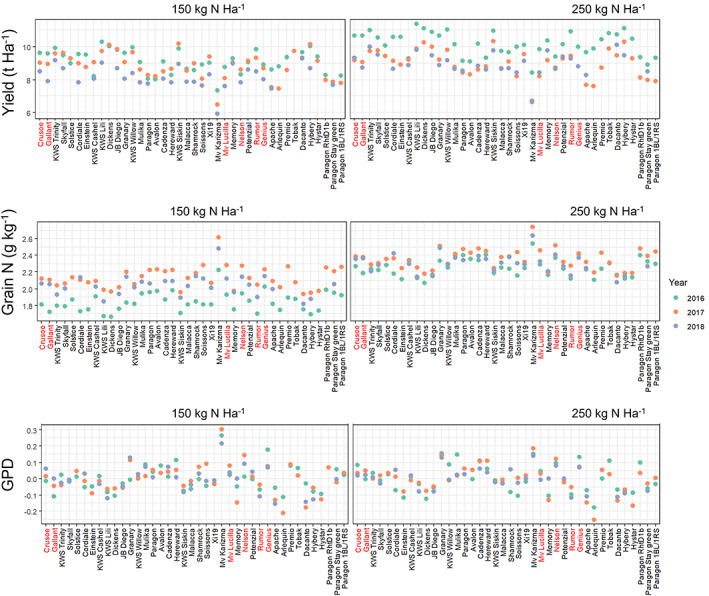
Means predicted by the linear mixed model for yield, grain N and grain nitrogen (% dry weight) and grain protein deviation (GPD) for the genotypes grown in 3 years. Names of selected lines are in red.

In general, there is a well established negative relationship between the yield and the concentration of protein in the wheat grain. However, some wheat genotypes consistently deviate from this relationship. This phenomenon is known as GPD and may be positive for high protein lines or negative for low protein lines.^14^ The results for yield, grain protein content (GPC) and GPD are therefore shown in Supporting Information, Table S3 and the predicted means in Fig. 1.

This showed clear differences between cultivars, which were broadly consistent between years. Variation in yield was to be expected as the cultivars included older and recent UK cultivars and other cultivars that were grown outside their area of adaptation. Hence, the Hungarian high protein cultivar Mv Karisma had the lowest yield and highest N content, whereas the modern UK cultivars generally had the highest yields.

Differences in grain N content were observed between cultivars, and between the N contents of the samples grown at N250 and N150. However, the extent of the latter differed between years, being greatest in 2016 and least in 2017. Finally, there were differences in the N content of samples from the different sites. This may have resulted from several factors: differences in residual soil N at the sites, differences in timings of fertiliser application (which followed the standard agronomic practices for the sites) and effects of other environmental factors.

### Milling and rheology

Based on these analyses, grain samples from the four median sites from each year were combined for milling. Low values for Hagberg Falling number in 2017 also resulted in the requirement to discard some samples, with Hereward being bulked from only two sites and Skyfall, Siskin, Shamrock, and Paragon Stay Green from only three sites. The yield of Mv Lucilla was particularly low in 2017 providing enough bulked grain (6 kg) for milling but not breadmaking.

The yields of white flours using a Bühler Laboratory Flour Mill MLU 202 ranged between 76.5% and 80.5% while water absorption (which is largely determined by starch damage during milling), measured using the Farinograph, gave typical values (means 56.8–59.1) for 2016, but unusually low values for 2017 (means 54.8–55.8) and 2018 (means 55.2–56.2) (Table 1).

**Table 1:** Mean flour yields and properties of white flours from all genotypes grown in the 3 years

	2016		2017		2018	
Nitrogen	150 kg Ha^‐1^	250 kg Ha^‐1^	150 kg Ha^‐1^	250 kg Ha^‐1^	150 kg Ha^‐1^	250 kg Ha^‐1^
Extraction rate (%)	78.7	80.4	79.1	78.9	76.8	76.8
Water absorption (@14%)	56.8	59.1	54.8	55.8	55.2	56.2
Moisture (% as is)	14.6	14.9	14.9	15.0	14.4	14.4
Protein (% as is)	8.9	10.9	9.7	10.8	9.2	10.5
Resistance (BU)	227	217	325	340	323	351
Extensibility (cm)	16.5	18.7	19.7	20.9	17.1	19.6
R/E (BU mm^‐1^)	1.4	1.2	1.6	1.6	1.9	1.8

**Figure 2:**
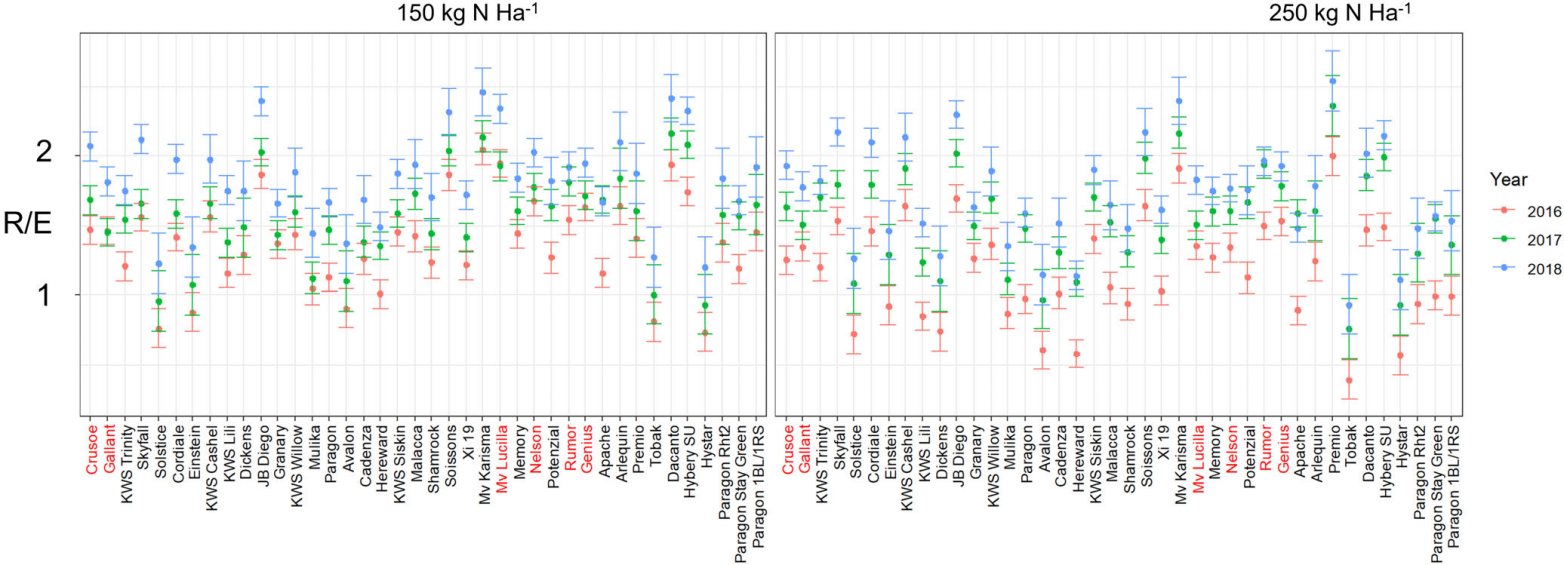
Means predicted by the linear mixed model and standard errors of R/E (dough elasticity) determined by Extensograph for the cultivars, predicted and averaged over the 3 years. Names of selected lines are in red. Years are 2016 (red), 2017 (green) and 2018 (blue).

Dough rheology was determined using a Brabender Extensograph. This measures the resistance (R) and extensibility (E) of dough, with R/E representing the balance between these properties. In broad terms, dough with R/E below 1.3 is too weak for breadmaking unless the protein content is very high. Dough with R/E between 1.3 and 1.7 is of moderate quality. Dough with R/E between 1.7 and 2.6 is of good quality, and dough with R/E above 2.6 is too strong for most UK breadmaking processes. In the present sample sets, R/E peaked between 1.5 and 2.0, but increased from 2016 to 2018 and was greater in the N250 samples (Fig. 2). Similar increases in R/E from 2016 to 2018 were observed when the full datasets (40 cultivars in 2016, 30 in 2017 and 20 in 2018) and only the 20 cultivars grown in all 3 years were considered (Supporting Information, Fig. S1A,B), indicating that they were related to the year and did not result from selection for quality over the 3 years.

Analysis of R/E (Supporting Information, Table S4) showed evidence of a significant interaction between N and variety. There is substantial variation, as estimated by variance components, between years and the interaction between year and N, but the variety by year interaction is estimated to be much smaller.

### Mixing and baking

The mixing properties and breadmaking performance of the white flours were determined by six milling and baking companies using their ‘in‐house’ quality testing systems. These included three different breadmaking systems: the Chorleywood Bread Process (CBP) (three processors), spiral mixing (two processors), and traditional fermentation (one processor). The 40 N250 samples grown in 2016 were analysed by two companies (using CBP and spiral) and the 40 samples N150 samples by four companies (two CBP, one spiral, and one traditional fermentation). The N250 and N150 samples of the 30 lines selected in 2017 were each analysed by five companies (three CBP, two spiral), and the N250 and N150 samples of the 20 lines selected in 2018 were each analysed by three companies (two CBP, one spiral). Hence, the total numbers of individual samples of each line that were analysed varied from six (four × N150 and two × N250 in 2016 only) to 22 (12 × N150 and 10 × N250, in 2016, 2017 and 2018 combined) (Supporting Information, Table S5). The use of three breadmaking processes, which also differed in detailed conditions between companies, and multiple samples therefore allowed the broad quality of the genotypes to be compared when grown at N250 and N150.

**Figure 3:**
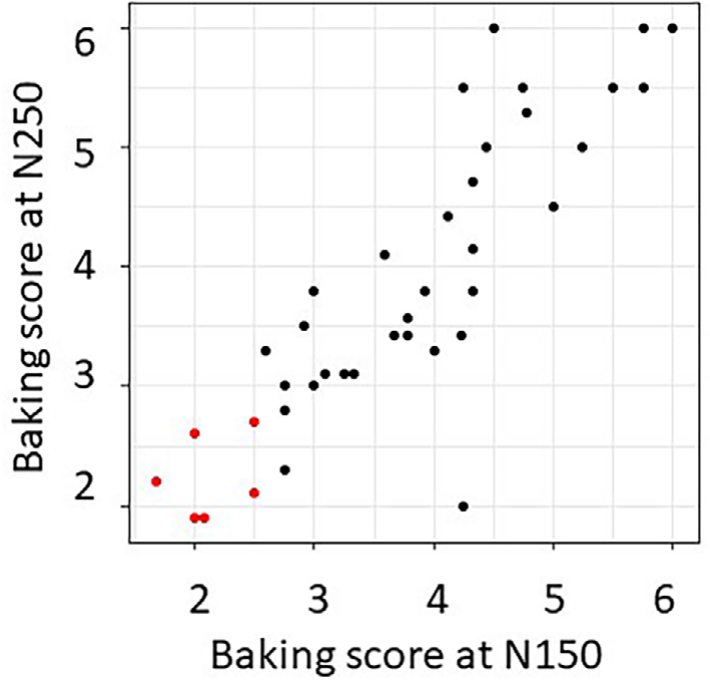
Correlations between breadmaking scores for N150 and N250 samples. Data are cultivar means (averaged over years and replicate analyses). Breadmaking scores were assigned by bakers based on the overall performance of the flour using their in‐house test baking system. These included loaf volume and/or baked height and crumb structure, colour and texture (see Supporting Information, Table S2). Selected lines are shown in red.

Multiple parameters were measured (Supporting Information, Table S2) allowing an overall ‘quality score’ to be assigned by each baker to each flour sample: these ranged from 1 (best quality) to 6 (lowest quality). There was good correlation between the breadmaking scores assigned for the N150 and N250 samples of the cultivars, averaged over sites and years (Fig. 3). Further analyses therefore focused on the N150 subset.

Comparison of the N150 samples showed that all cultivars varied widely in their scores between years, sites and processes, with scores ranging from 1 to 4, 5 or 6 (Table 2). Nevertheless, six cultivars clearly performed better at N150 with mean scores for 12 breadmaking analyses of 2.5 or below. These were Crusoe and Gallant, which are current UK UKFM group 1 cultivars, Rumor and Nelson which are German varieties bred to show high quality at low grain protein and Genius which is a German breadmaking cultivar. In addition, Mv Lucilla (a Hungarian breadmaking wheat with high grain protein content) also had a low score (2.0) based on seven baking analyses of the 2016 and 2018 samples.

**Table 2:** Breadmaking scores for the cultivars grown at N150. The five cultivars that consistently gave good breadmaking scores at 150 kg N Ha^‐1^ (means of 2.5 or below) are shown in bold. Breadmaking scores were assigned by bakers based on the overall performance of the flour using their in‐house test baking system. These included loaf volume and/or baked height and crumb structure, colour and texture (see Supporting Information, Table S2).

		Number of breadmaking scores			*n*	Breadmaking scores			
Type	Cultivar	2016	2017	2018		Mean	SD	Min.	Max.
UKFM 1 Winter type	**Crusoe**	**4**	**5**	**3**	**12**	**1.67**	**1.23**	**1**	**4**
	**Gallant**	**4**	**5**	**3**	**12**	**2.08**	**1.56**	**1**	**6**
	KWS Trinity	4	5	3	12	2.75	1.91	1	6
	Skyfall	4	5	3	12	3.58	2.02	1	6
	Solstice	4	0	0	4	4.75	0.957	4	6
UKFM 2 Winter type	Cordiale	4	5	3	12	3.25	1.6	1	6
	Einstein	4	0	0	4	5.25	0.957	4	6
	KWS Cashel	4	5	0	9	4.33	1.66	1	6
	KWS Lili	4	5	3	12	3.33	1.92	1	6
UKFM 4 Winter type	Dickens	4	0	0	4	5	2	2	6
	JB Diego	4	5	3	12	4.33	1.3	2	6
UK Spring type	Granary	4	5	3	12	3.25	1.66	1	6
	KWS Willow	4	5	0	9	3.78	1.92	1	6
	Mulika	4	5	0	9	3.67	2.18	1	6
	Paragon	4	5	3	12	2.75	2.01	1	6
Older UK breadmaking	Avalon	4	0	0	4	5.75	0.5	5	6
	Cadenza	4	5	0	9	3.78	1.86	1	6
	Hereward	4	5	3	12	3	1.54	1	6
	KWS Siskin	4	5	3	12	4	1.81	1	6
	Malacca	4	5	0	9	3	1.41	1	5
	Shamrock	4	5	0	9	4.33	1.22	3	6
	Soissons	4	5	0	9	4.44	1.67	1	6
	Xi 19	4	5	3	12	3.08	1.78	1	6
Hungarian high protein	Mv Karisma	4	5	0	9	4.78	1.3	2	6
	**Mv Lucilla**	**4**	**0**	**3**	**7**	**2**	**1.41**	**1**	**4**
German low protein breadmaking	Memory	4	5	3	12	2.92	1.93	1	6
	**Nelson**	**4**	**5**	**3**	**12**	**2.5**	**1.45**	**1**	**5**
	Potenzial	4	5	0	9	4.22	1.79	1	6
	**Rumor**	**4**	**5**	**3**	**12**	**2.5**	**1.83**	**1**	**6**
German breadmaking	**Genius**	**4**	**5**	**3**	**12**	**2**	**1.41**	**1**	**5**
French breadmaking	Apache	4	5	3	12	2.58	1.56	1	5
	Arlequin	4	0	0	4	5.5	0.577	5	6
	Premio	4	0	0	4	5.75	0.5	5	6
	Tobak	4	0	0	4	6	0	6	6
Danish breadmaking	Decanto	4	5	0	9	4.11	1.36	1	6
Hybrid wheats	Hybery	4	5	3	12	3.92	1.62	1	6
	Hystar	4	0	0	4	4.5	1.73	2	6
Paragon lines	Paragon Rht2	4	0	0	4	4.25	1.26	3	6
	Paragon Stay Green	4	5	3	12	2.75	2.09	1	6
	Paragon 1BL/1RS	4	0	0	4	4.25	2.36	1	6

### Size‐Exclusion High‐Performance Liquid Chromatography

**Figure 4:**
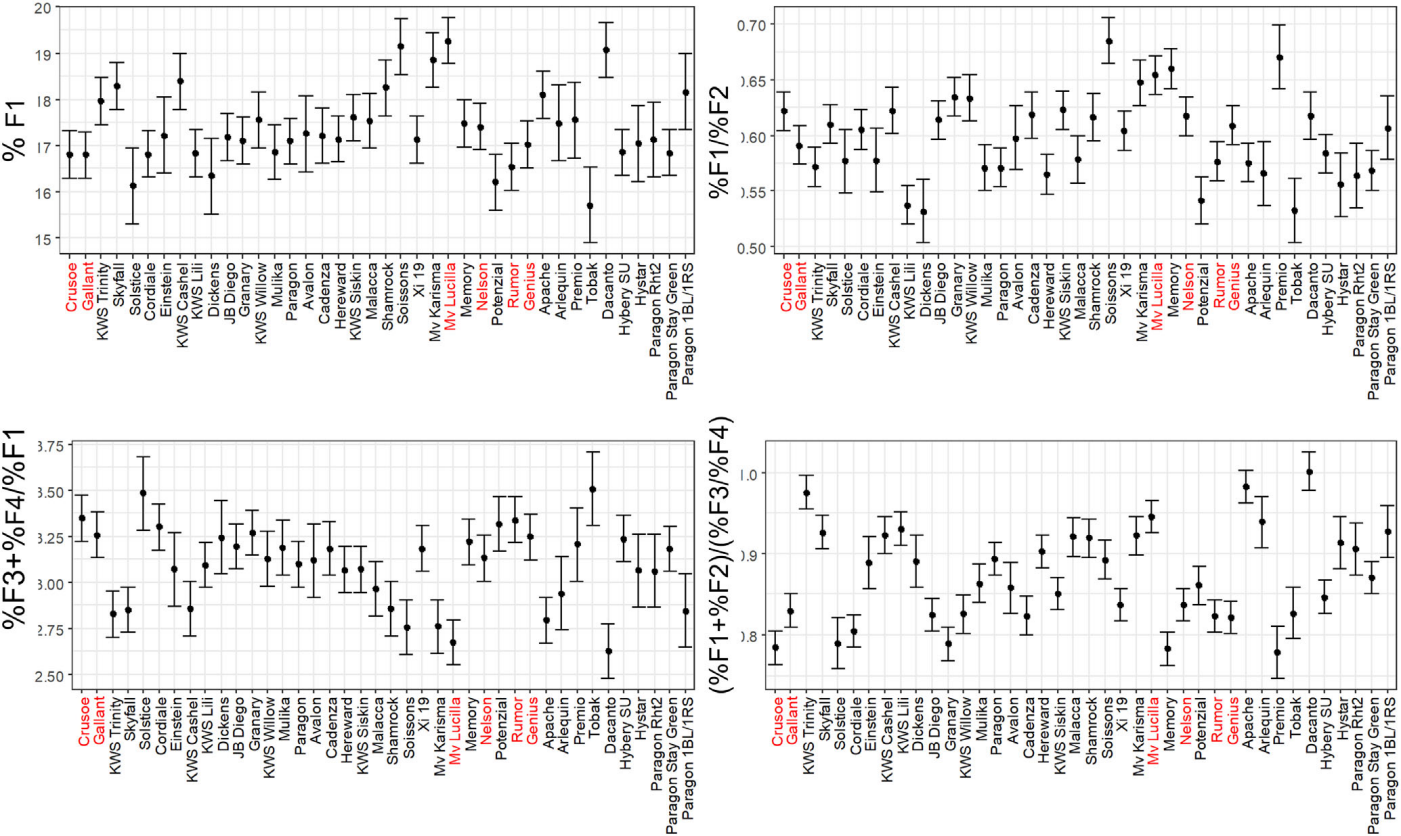
Proportions of peaks determined by Size‐Exclusion High‐Performance Liquid Chromatography (SE‐HPLC) of protein extracts from the N150 samples over the 3 years. Predicted means, averaged over the 3 years and associated standard errors are shown. Selected lines are shown in red.

Size‐Exclusion High‐Performance Liquid chromatography was carried out on all N150 samples to determine the size distribution of gluten proteins (glutenin polymers and gliadin monomer). Millar^16^ showed that accurate estimates of dough strength were provided by comparing the ratio of large to small glutenin polymers (%F1/%F2) and the ratio of gliadins to large glutenin polymers (%F3 + %F4)/(%F1), and data for these parameters are shown in Fig. 4. Data for (%F1 and %F1 + %F2)/(%F3 + %F4) are also shown, as these measure the proportion of HMW glutenin polymers and the glutenin:gliadin ratio, respectively, both of which have been used as measures of quality. Comparison of the genotypes showed wide variation in the proportions and ratios of peaks. In particular, the %F1 (large glutenin polymers) ranged from below 16% to above 19% and the (%F1 + %F2)/(%F3 + %F4) (glutenin:gliadin ratio) from below 0.8 to about 1.0. Analysis showed that there was a highly significant effect of genotype for all parameters (Supporting Information, Table S6).

### Statistical comparison of selected and non‐selected cultivars

To identify differences between the six selected cultivars and the remaining unselected cultivars a comparison was carried out focusing on four parameters: grain N determined by NIRS of samples from individual field plots as a measure of grain protein (N × 5.7 = protein), GPD, the SE‐HPLC profiles and R/E measured by the Extensograph. The means and standard errors for the selected and non‐selected groups and statistical comparisons of the two groups are given in Table 3.

**Table 3:** Means, standard errors, and statistical comparisons between selected and non‐selected genotypes at N150, resulting from approximate *F*‐tests using Kenward–Roger denominator degrees of freedom from the mixed models as described in the Materials and Methods section. Significant *P* values are shown in bold.

	Selected		Non‐selected		Comparison of selected and non‐selected genotypes			
Trait	Mean	SE	Mean	SE	*ndf*	*ddf*	*F* statistic	*P*‐value
Grain N (g kg^−1^)	2.029	0.0062	1.99	0.0027	1	1488.5	28.19	**<0.001**
GPD	+0.02277	0.00617	−0.00446	0.00273	1	1489.7	16.34	**<0.001**
R/E	1.791	0.043	1.535	0.022	1	48	14.68	**<0.001**
%F1	17.31	0.1829	17.38	0.0915	1	48.3	0.35	0.555
%F1/%F2	0.6115	0.0065	0.5963	0.0032	1	48.3	3.73	0.059
%F3 + %F4/%F1	3.166	0.044	3.087	0.022	1	48.3	3.61	0.063
%F1 + %F2/%F3 + %F4	0.8402	0.0070	0.8747	0.0035	1	48.2	23.31	**<0.001**

Statistical analysis showed significant differences between the groups of selected and non‐selected cultivars with percentage grain N (ie. GPC), GPD and R/E all being higher in the group of selected cultivars. By contrast, %F1 + %F2 / %F3 + %F4 (glutenin:gliadin ratio) was significantly lower in the group of selected cultivars, with no significant differences for %F1/%F2 (ratio of large to small glutenin polymers), %F3 + %F4 / %F1 (ratio of gliadins to large glutenin polymers) and %F1 (% large glutenin polymers) (Table 3).

When considered as a group, therefore, the six cultivars that performed well at N150 had higher grain N (protein), GPD and dough elasticity (R/E) than the non‐selected cultivars. Hence, good performance with reduced N fertiliser resulted from two factors: efficient translocation of N into the grain and increased dough elasticity (strength).

## DISCUSSION

We have compared the compositions and breadmaking quality of 40 genotypes of bread wheat when grown under optimal (250 kg N Ha^‐1^) and suboptimal (150 kg N Ha^‐1^) levels of N fertilisation. The genotypes were selected to include a range of modern cultivars bred for the UK as well as a wider range of genotypes including Hungarian cultivars bred for high grain protein and German cultivars bred for good processing quality under low N fertilisation. Although all 40 cultivars were grown on six sites for 2 years, only 30 were grown for a third year, eliminating cultivars which were poorly adapted to the UK. Similarly, analyses of grain composition and quality were carried out on subsets of 30 and 20 cultivars from years 2 and 3, respectively.

Comparisons of the breadmaking performance of white flours were made by six commercial grain quality laboratories, using three processes. The combination of quantitative measures of mixing and breadmaking quality with subjective assessments of quality by highly experienced bakers allowed the grain samples to be assigned ‘breadmaking scores’ from 1 (best) to 6 (poorest).

This identified six cultivars that gave good performance at N150: Crusoe, Gallant (both AHDB Group 1), Rumor, Nelson (both German varieties bred for high quality at low grain protein), Genius (German breadmaking wheat) and Mv Lucilla (Hungarian high protein bread making cultivar).

Both Crusoe and Gallant were highly successful breadmaking cultivars, widely grown in the UK. The study therefore shows that modern cultivars, which have been selected for performance in high input systems, may also perform well under lower N inputs. It is also notable that Crusoe has the ‘dicoccoides’ chromosome introgression associated with higher grain protein content. However, it should be noted that only one of the non‐UK cultivars, Rumor, had a comparable yield to the modern UK cultivars (Crusoe, Gallant), while Mv Lucilla and Nelson were among the lowest yielding (Fig. 1).

The selected cultivars had statistically significantly higher grain N and strong positive GPD compared with the non‐selected cultivars. The correlation of GPD with good breadmaking quality at reduced levels of N application is not surprising, as GPD has long been recognised as an important factor contributing to the efficiency of N use in wheat with positive GPD indicating a better ability to partition N to the grain for any given yield.^8^, ^17^, ^18^ A detailed study of GPD in the samples reported here quantified GPD in the individual cultivars and calculated the heritability as 0.44, with genotype making the largest contribution to this (0.33).^14^ The proportion of gluten proteins increases with increasing grain protein content (reviewed by Shewry^19^) and GPD therefore results in a higher content of gluten proteins.

The association between good breadmaking quality at low protein with high dough elasticity (R/L) measured by Extensograph is consistent with our understanding of the relationship between dough rheology and processing quality.

Statistical comparison showed that the group of selected cultivars had a slightly lower proportion of glutenin polymers (measured as [%F3 + %F4]/[%F4 + %F5]). However, the difference was small (0.84 compared with 0.87), with both values being lower than in a previous study that used the same methodology (from 0.978 to 1.161).^20^ This suggests that the higher protein content was more important in determining the breadmaking quality of the selected cultivars than glutenin polymer content.

The high protein cultivar Mv Lucilla differed from the other five selected cultivars in having higher proportions of total glutenin ([%F3 + %F4]/[%F1 + %F2]) and large glutenin polymers (%F1) and a low ratio of gliadins to large glutenin polymers (%F3 + F4/%F2) (Fig. 4). However, Mv Lucilla was poorly adapted to the UK, with low yields and low Hagberg falling number. Hence, in this case, the increased dough strength conferred by the high glutenin content and large glutenin polymers may have compensated for deficiencies in other aspects of quality.

The work reported here therefore shows that breeding for high grain protein, particularly by exploiting GPD, combined with high gluten elasticity, will contribute to the development of cultivars with good breadmaking performance when grown with reduced levels of N fertiliser. However, it should be noted that the differences between the individual selected cultivars and non‐selected cultivars were relatively small and not all of the selected cultivars exhibited both GPD and high dough strength (as shown in Figs 1, 2 and 4 and reported for GPD by Mosleth *et al*.^14^). Nevertheless, the study shows that selection for high grain protein and higher dough elasticity should allow the development of cultivars with better breadmaking quality at lower levels of nitrogen fertilisation. Furthermore, these cultivars should also perform well when grown with higher levels of N fertiliser, meaning that separate breeding programmes will not be required.

## Supporting information


**Data S1.** Supporting information.

## Data Availability

Full datasets available from the corresponding author
