# Supplementary material for: Identification of traits underpinning good breadmaking performance of wheat grown with reduced nitrogen fertilisation
Source: J Sci Food Agric. 2023 Jul 26;103(15):7664–72. doi: 10.1002/jsfa.12848 (PMC10952534; doi:10.1002/jsfa.12848)
Supplement: Supplementary file 1 — Data S1. Supporting information. [file JSFA-103-7664-s001.docx]

**Supplementary Material**

A

B

**
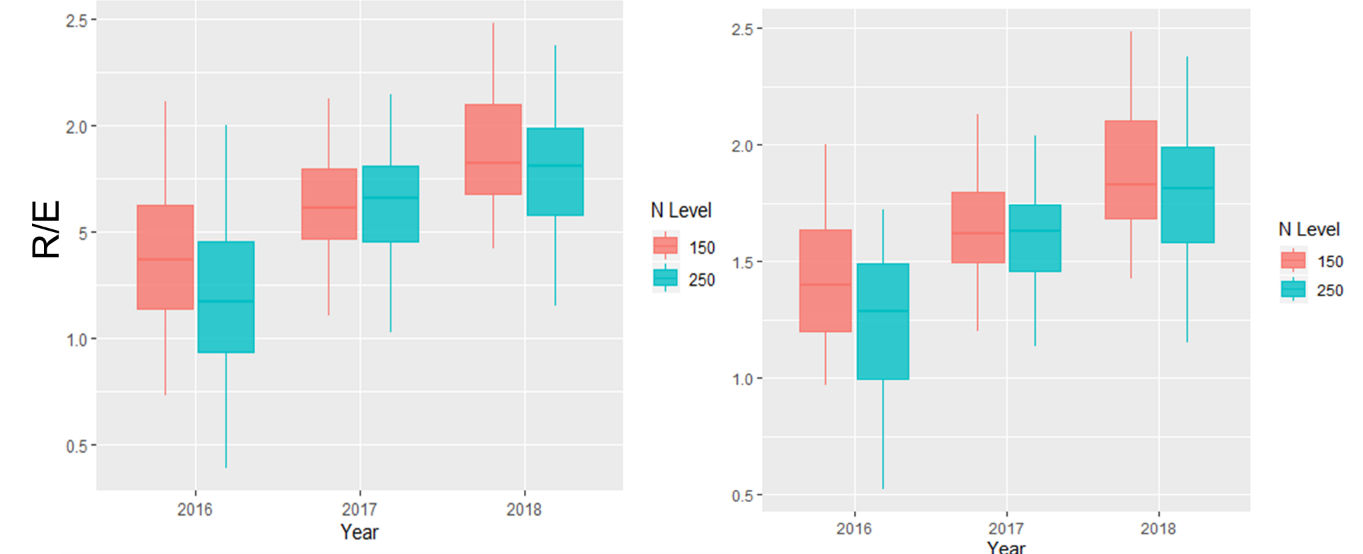
**

**Figure S1.** Dough elasticity (R/E) determined by Extensograph

A, for all cultivars grown and analysed in 2016, 2017 and 2018

B, for the 20 cultivars grown and analysed in all three years

The individual cultivars are colour coded in Supplementary **Table S1** below.

**Table S1**. Wheat genotypes selected for field trials

Genotypes which were grown and analysed in all three years (2015-6, 2016-7, 2017-8) are shown in black. Genotypes which were grown in 2015-6 and 2016-7 but not analysed from 2016-7 are shown in red. Samples that were grown in all 3 years but not analysed from 2017-8 are shown in green.

| **Type** | **Cultivar** | **Type** | **Cultivar** |
| --- | --- | --- | --- |
| UKFM 1  Winter type | Crusoe | Older UK breadmaking | Shamrock |
|  | Gallant |  | Soissons |
|  | KWS Trinity |  | Xi 19 |
|  | Skyfall | Hungarian high protein | Mv Karisma |
|  | Solstice |  | Mv Lucilla |
| UKFM 2  Winter type | Cordiale | German low protein breadmaking | Memory |
|  | Einstein |  | Nelson |
|  | KWS Cashel |  | Potenzial |
|  | KWS Lili |  | Rumor |
| UKFM 4  Winter type | Dicken | German breadmaking | Genius |
|  | JB Diego | French breadmaking | Apache |
| UK Spring type | Granary |  | Arlequin |
|  | KWS Willow |  | Premio |
|  | Mulika |  | Tobak |
|  | Paragon | Danish breadmaking | Decanto |
| Older UK breadmaking | Avalon | Hybrid wheats | Hybery |
|  | Cadenza |  | Hystar |
|  | Hereward | Paragon lines | Paragon Rht2 |
|  | KWS Siskin |  | Paragon Stay Green |
|  | Malacca |  | Paragon 1BL/1RS |

**Table** **S.2**. Mixing, baking and loaf quality parameters measured by milling and baking companies using their “in house” procedures

| **Mixing and baking** | **Loaf and crumb** |
| --- | --- |
| Mixing time  Dough temperature  Dough strength  Dough extensibility  Dough handling  Proof height | Loaf volume and/or baked height  Crumb colour  Crumb texture  Crumb structure |

**Table S3.** Results from the linear mixed model analysing yield, grain N and GPD for the 30 genotypes grown over 3 years and 10 genotypes grown for two years.

Shown are the denominator degrees of freedom (ddf), approximate Kenward-Roger F-statistic and associated p-values. Where the highest order terms were not significant, they were dropped from the fitted model. To ensure convergence of the REML algorithm when fitting GPD, random effect strata were combined inflating the ddf associated with the effect of time.

| **Term** | **Yield** | | | **Grain N** | | | **GPD** | | |
| --- | --- | --- | --- | --- | --- | --- | --- | --- | --- |
|  | ddf | F | **p** | **ddf** | **F** | **p** | **ddf** | **F** | **p** |
| Time | 7.8 | 0.94 | 0.430 | 8.0 | 3.79 | 0.069 | 44 | 0 | 1 |
| Cultivar | 3087.4 | 75.5 | <0.001 | 3048.7 | 105.9 | <0.001 | 2981.7 | 50.4 | <0.001 |
| N | 41.6 | 75.3 | <0.001 | 42.1 | 406.0 | <0.001 | 42.6 | 0 | 0.99 |
| Time.Cultivar |  |  | ns | 3048.7 | 6.15 | <0.001 | 2981.7 | 4.11 | <0.001 |
| Time.N | 41.6 | 13.05 | <0.001 | 42.3 | 15.4 | <0.001 | 43.8 | 0 | 1 |
| Cultivar.N | 3087.5 | 5.73 | <0.001 | 3048.8 | 3.35 | <0.001 | 2981.7 | 3.07 | <0.001 |
| Time.Cultivar.N |  |  | ns |  |  | ns | 2981.7 | 1.37 | <0.001 |

*ns: not significant, **HOT: higher order term included in in the model

**Table S4.** Results from the linear mixed model of R/E measured by Extensograph.

Shown are the denominator degrees of freedom (ddf), approximate Kenward-Roger F-statistic and associated p-values. Since samples were pooled across all replicates at all sites, the fixed effects below were tested having fitted random effects to Year (estimated variance component 0.06) and the interaction terms Year.N(estimated variance component 0.004) and Year.Cultivar (estimated variance component 0.01).

| Term | ddf | F-statistic | p-value |
| --- | --- | --- | --- |
| N | 2 | 3.69 | 0.197 |
| Cultivar | 48 | 8.34 | <0.001 |
| Cultivar. N | 48.1 | 1.87 | 0.020 |

**Supplementary Table S5.** Numbers of samples analysed for breadmaking quality.

| **Type** | **Cultivar** | **Number of scores by years** | | | **Number of scores by N level** | |
| --- | --- | --- | --- | --- | --- | --- |
|  |  | **2016** | **2017** | **2018** | **150** | **250** |
| UKFM 1  Winter type | Crusoe | 6 | 10 | 6 | 12 | 10 |
|  | Gallant | 6 | 10 | 6 | 12 | 10 |
|  | KWS Trinity | 6 | 10 | 6 | 12 | 10 |
|  | Skyfall | 6 | 10 | 6 | 12 | 10 |
|  | Solstice | 6 | 10 | 0 | 9 | 7 |
| UKFM 2  Winter type | Cordiale | 6 | 10 | 6 | 12 | 10 |
|  | Einstein | 6 | 0 | 0 | 4 | 2 |
|  | KWS Cashel | 6 | 10 | 0 | 9 | 7 |
|  | KWS Lili | 6 | 10 | 6 | 12 | 10 |
| UKFM 4  Winter type | Dicken | 6 | 0 | 0 | 4 | 2 |
|  | JB Diego | 6 | 10 | 6 | 12 | 10 |
| UK Spring type | Granary | 6 | 10 | 6 | 12 | 10 |
|  | KWS Willow | 6 | 10 | 0 | 9 | 7 |
|  | Mulika | 6 | 10 | 0 | 9 | 7 |
|  | Paragon | 6 | 10 | 6 | 12 | 10 |
| Older UK breadmaking | Avalon | 6 | 0 | 0 | 4 | 2 |
|  | Cadenza | 6 | 10 | 0 | 9 | 7 |
|  | Hereward | 6 | 10 | 6 | 12 | 10 |
|  | KWS Siskin | 6 | 10 | 6 | 12 | 10 |
|  | Malacca | 6 | 10 | 0 | 9 | 7 |
|  | Shamrock | 6 | 10 | 0 | 9 | 7 |
|  | Soissons | 6 | 10 | 0 | 9 | 7 |
|  | Xi 19 | 6 | 10 | 6 | 12 | 10 |
| Hungarian high protein | Mv Karisma | 6 | 10 | 0 | 9 | 7 |
|  | Mv Lucilla | 6 | 0 | 6 | 7 | 5 |
| German low protein breadmaking | Memory | 6 | 10 | 6 | 12 | 10 |
|  | Nelson | 6 | 10 | 6 | 12 | 10 |
|  | Potenzial | 6 | 10 | 0 | 9 | 7 |
|  | Rumor | 6 | 10 | 6 | 12 | 10 |
| German breadmaking | Genius | 6 | 10 | 6 | 12 | 10 |
| French breadmaking | Apache | 6 | 10 | 6 | 12 | 10 |
|  | Arlequin | 6 | 0 | 0 | 4 | 2 |
|  | Premio | 6 | 0 | 0 | 4 | 2 |
|  | Tobak | 6 | 0 | 0 | 4 | 2 |
| Danish breadmaking | Decanto | 6 | 10 | 0 | 9 | 7 |
| Hybrid wheats | Hybery | 6 | 10 | 6 | 12 | 10 |
|  | Hystar | 6 | 0 | 0 | 4 | 2 |
| Paragon lines | Paragon Rht2 | 6 | 0 | 0 | 4 | 2 |
|  | Paragon Stay Green | 6 | 10 | 6 | 12 | 10 |
|  | Paragon 1BL/1RS | 6 | 0 | 0 | 4 | 2 |

**Table S6**. REML analysis of F1/F2 and F1+F2/F3+F4 for the low N samples grown in three years through linear mixed models. Table shows the approximate (Kenward-Roger) F-statistic for the variety fixed effect when year is included as a random effect. The year.variety term is the residual.

| Variable | ndf | ddf | F statistic | p-value |
| --- | --- | --- | --- | --- |
| F1 | 39 | 48.1 | 2.3 | 0.003 |
| F1/F2 | 39 | 48.1 | 3.71 | <0.001 |
| F3+F4/F1 | 39 | 48.1 | 2.78 | <0.001 |
| F1 + F2 / F3 + F4 | 39 | 48.1 | 9.27 | <0.001 |
